# Our New York Letter

**Published:** 1892-02

**Authors:** 


### Correspondence.

### NEW YORK LETTER.

NEW YORK, January 22, 1892.

Small-pox has made its appearance in this city. It seems to have taken root in the Italian colony, at Mulberry street, its source having been directly traced to New Jersey, where the disease has raged with more or less severity for several weeks. The whole of the Health Board corps of vaccinators are hard at work vaccinating all the residents of the Italian colony, and it is expected that over 10,000 persons will be vaccinated within the next few days.

Six cases of small-pox have been discovered within the past few days, all among the Italians of New York, and the houses where these have been living have been thoroughly fumigated, while the bedding on which the patients lay has been destroyed. All the neighboring tenement houses, which swarm with Italians, have been treated in a similar manner, while hourly inspections of the persons remaining in the infected tenements are being made by the medical corps.

At last night's meeting of the section of hygiene at the Academy of Medicine, Dr. A. L. Loomis, speaking of the contagiousness of pulmonary tuberculosis, said: "Tuberculosis must be classed among contagious diseases. The most important question before this meeting is to stop the spread of this contagion. To this end, the first step is to educate the public in this new matter of contagion, to let them know how contagious tuberculosis is, and how it can be prevented. The key-note to this knowledge is cleanliness and out-of-door life. Give the poor more and more fresh air to breathe; give them anything that will make them clean, and, at the same time, educate them about the contagiousness of tuberculosis."

Dr. A. G. Gerster, of the New York Polyclinic, presented recently to the class a young man with chronic inflammation of the deep urethra. He came to the clinic suffering from frequent urination, passing his water every ten or fifteen minutes. He was given four deep urethral injections of a five per cent. solution of nitrate of silver, and at the end of that time was able to retain his water without difficulty for a period of six hours, all the other general symptoms of chronic urethritis disappearing at the same time.

The injections are made after the patient has urinated. He is then placed on his back and the instrument introduced beyond the cut-off muscle and inserted in the neck of the bladder, but not into the bladder. It must lie in that space situated between the cut-off muscle and the vesical sphincter, as it is in this portion of the urethra that the trouble is situated. Three or five minims of a five per cent. solution of nitrate of silver should be injected once, twice or three times a week, according to the urgency of the case.

Dr. Gerster says that he has seen well informed physicians attempt to cure cases of chronic urethritis of this kind by the indroduction of full-sized sounds.

"You may, by these means," he adds, "aggravate the trouble, but you cannot cure it." In the treatment of urethral diseases, he thinks we must be guided by a clear understanding of the exciting condition. It is a subject that is much neglected, but is one that forms a good proportion of the daily practice of many physicians, and is one well worthy of their attention.

The following is the method adopted at Roosevelt Hospital for the treatment of incomplete abortion. The uterus is emptied of the fœtus as soon as possible, and if the hemorrhage is not profuse, the patient is given a full bath. The extraction is done with all the precautions taken in laparotomy.

The external genitals are thoroughly scrubbed and washed with bi-chloride solution, the patient placed in the lithotomy position, the vagina fornix and cervix scrubbed with soap and water, and disinfected with bi-chloride. The cervix is then cleansed of mucus and washed. An assistant next holds the perineum back, with a speculum, while another pulls down the cervix with a tenaculum, and with the uterus grasped and held through the abdominal walls, the surgeon proceeds, if the cervix is not sufficiently dilated, to dilate it with a metal dilator.

The uterus is then emptied of the ovum, membranes and shreds, by means of the finger, the forceps and curette.

It is then irrigated with bi-chloride solution, and if contraction does not take place and arrest the hemorrhage, tincture of iodine is applied, and in neglected cases, a tamponade of iodoform gauze is introduced, which is gradually removed during the twenty-four hours, commencing after the third hour.

The bladder is emptied by a catheter every twenty-four hours. The patient usually remains a week in the hospital.

The Cartwright Lectures for 1892 were delivered by Prof. Henry F. Osborn, of Columbia College, at the New York Academy of Medicine, at 8 P. M., from February 12th to 19th, 1892. The subject of the lectures was "The Present Problem in Evolution and Heredity." They were listened to by a large and appreciative audience, and were well received.

A large number of out-of-town doctors are at present visiting New York, and the South, it may be safely said, is well represented. There is no end to the operations, clinics, lectures, and discussions on medical subjects that take place in this city every day of the week. Go to the most out of the way clinic, and select even the most disagreeable day for your observation, and you will be surprised to see the large numbers of physicians in attendance from out of town. These are not by any means the pinion members of the profession either, but old and experienced men whose enthusiasm and earnestness for their calling seem to have increased with age. The surgical clinics are the ones most frequently visited, and perhaps no other field in the world can furnish an equal number of interesting surgical cases than can New York. After the work of the day is over these same gentlemen may be seen in the Academy of Medicine listening to discussions with the same eagerness and attention, and the papers that are read in the Academy are frequently excellent in every respect. These discussions are sometimes kept up to a very late hour at night and those who have come to listen to them rarely go away disappointed.

